# Preparing for resilience — Si Vis Pacem, Para Bellum

**DOI:** 10.17269/s41997-025-01106-5

**Published:** 2025-09-11

**Authors:** Victoria Haldane, Andrew Beckett, Paul Engels, Colleen Forestier, David Gomez, David Klein, David Pedlar, Manveen Puri, David Redpath, Anthony Robb, Adalsteinn Brown

**Affiliations:** 1 Dalla Lana School of Public Health, University of Toronto, 155 College St Room 500, Toronto, ON Canada; 2 Department of Surgery, University of Toronto, 149 College Street, 5th Floor, Toronto, ON Canada; 3 Canadian Forces Health Services, 713 Montréal Rd, Gloucester, ON Canada; 4 Faculty of Health Sciences, McMaster University, 1280 Main Street West, Hamilton, ON Canada; 5 Unity Health, 61 Queen St E, Toronto, ON Canada; 6 University of Ottawa, 75 Laurier Ave E, Ottawa, ON Canada; 7 Department of Medicine, University of Toronto, 6 Queen’s Park Crescent West, Toronto, ON Canada; 8 Canadian Joint Operations Command, 1600 Star Top Rd, Ottawa, ON Canada

**Keywords:** Preparedness, Planning, Public health and health systems, Crisis management, All hazards, Complex emergencies, Tabletop exercise, Préparation, Planification, Systèmes de santé publique et de santé, Gestion de crise, Tous risques, Situations d’urgence complexes, Exercice sur table

## Abstract

Canada has faced numerous public health challenges but remains inadequately prepared for future crises. For example, despite extensive reports and plans following the 2003 SARS-CoV-1 outbreaks, the country was unprepared for COVID-19 and lessons learned from the pandemic emphasized the need for immediate action to enhance preparedness. In the current era of poly-crisis, Canada must be ready for diverse challenges, including potential conflicts and their impacts on public health and health systems. The conflict in Ukraine highlights the need for extensive medical resources for returnees, which could strain public health and health systems alongside other concurrent threats. Exercise Trillium Cura (ETC) in 2024 simulated Ontario’s health system response to a prolonged conventional war, revealing both successes and challenges. Key issues included leadership and resource needs, with recommendations for specific actions like creating a repatriation hub and a trauma registry. ETC emphasized a “whole of society” approach, engaging civil society in planning and highlighting the importance of integrated preparedness. Tabletop exercises like ETC are crucial for building relationships, shared learning, and innovative solutions. They help prepare for complex crises by fostering collaboration and readiness. Regular exercises are recommended to enhance preparedness and resilience, ensuring effective responses to future health emergencies.

## Résumé

Le Canada a affronté de nombreux problèmes de santé publique, mais il est encore insuffisamment préparé aux crises. Par exemple, malgré les rapports et les plans détaillés qui ont suivi les éclosions de SRAS-CoV-1 de 2003, le pays était mal préparé à la COVID-19, et les leçons tirées de la pandémie soulignent le besoin d’agir immédiatement pour renforcer cette préparation. À l’ère des polycrises, le Canada doit absolument être prêt pour divers problèmes, dont des conflits possibles et leurs effets sur les systèmes de santé publique et de santé. Le conflit en Ukraine montre qu’il faut consacrer des ressources médicales considérables aux personnes rapatriées, ce qui peut grever les systèmes de santé publique et de santé s’il y a d’autres menaces concurrentes. Mené en 2024, l’exercice Trillium Cura (ETC) a simulé la réaction du système de santé ontarien à une guerre conventionnelle prolongée pour en cerner les points forts et les points faibles. Les principaux points faibles ont été le manque de leadership et de ressources, et des mesures particulières ont été recommandées, comme l’établissement d’un centre de rapatriement et d’un registre des traumatismes. L’ETC a insisté sur une approche impliquant toute la société, avec la participation de la société civile à la planification, et souligné l’importance d’une préparation intégrée. Les exercices sur table comme l’ETC sont cruciaux pour resserrer les liens, partager les enseignements et trouver des solutions novatrices. Ils contribuent à préparer le système aux crises complexes en favorisant la collaboration et la préparation. Il est recommandé de mener périodiquement de tels exercices pour renforcer la préparation et la résilience, et assurer ainsi une réponse efficace aux futures urgences sanitaires.

## Failing to learn from crisis

Canada has faced a series of challenges to its public health and health systems but has yet to achieve adequate preparedness – an essential public health function. The 2003 SARS-CoV-1 outbreaks in Canada were followed by recommendation-rich reports and preparedness plans at the provincial and federal levels (Public Health Agency of Canada, 2004; The Honourable Archie Campbell, 2006). Despite these efforts, Canada was again unprepared in many ways for the far-reaching impacts of the COVID-19 pandemic. The only federal review of the COVID-19 pandemic, led by Sir Mark Walport on our scientific readiness, again emphasized that “Canada needs to take further action now if it is to be adequately prepared for future health emergencies” (Health Canada, 2024). We are not ready for the next health emergency, but there are steps we can take today to improve our preparedness and resilience to withstand the broad range of changes we face for readiness.

In the age of the poly-crisis, a term coined by Edgar Morin in 1999, we need to be prepared for a wider range of challenges to our public health and health systems and how each one can amplify the impact of the next (Morin & Kern, 1999). High-level reports consistently underscore that conflict and war are global mega-trends that will impact public health and health systems in the coming years (European Commission, 2023; Policy Horizons Canada, 2024; World Health Organization, 2022). Indeed, the growing likelihood of a general war with a peer adversary, and the possibility of use of non-conventional weapons, whether in Eastern Europe or elsewhere, demands we consider the realities of this challenge. Like the First and Second World Wars, many of our wounded, as well as non-combatants and even prisoners of war, will be evacuated to Canada in need of care. Canada has no dedicated military inpatient hospital or rehabilitation beds. The brutal nature of the conflict in Ukraine, with its deployment of new types of weapons leading to more devastating injury patterns, suggests that these returnees will need extensive critical, surgical, burn, rehabilitation and mental health care resources (Anderson, 2023). Even a conservative application of the all-hazards approach means that our public health and health systems may have to cope with these added pressures while also dealing with concurrent threats including heat domes, wildfires, floods, and other challenges that could persist for years. These extra demands could easily and quickly exceed those seen during the COVID-19 pandemic, limiting our ability to carry out essential public health functions like health protection and promotion, while also undermining our ability to provide care. Casualties from the conflict would flood our hospitals, forcing the postponement or cancelation of necessary care for civilian and military patients, and damaging the promise of our system to care for Canadians, and the particular duty to care for our wounded.

## Exercise Trillium Cura

In 2024, a collaboration across the academic, health care, public health, public service and the Canadian Armed Forces launched Exercise Trillium Cura (ETC) (Exercise Trillium Cura Post-Exercise Report, 2004). ETC was a tabletop simulation of how the health system in Ontario, along with a wide range of partners, would cope with the inflow of casualties from a prolonged conventional war while maintaining care for civilians. The result of six months of planning and development by a civilian-military team, Trillium Cura brought together 45 in-person participants and 27 observers from across the country using wargame techniques to simulate the flow of a wide-range of wounded into Ontario while maintaining current clinical volumes. Baseline estimates derived from multiple sources, ETC assumed an average of 45 patients per week needing triage and onward care and management from the Ontario health system (Gomez et al., 2025). ETC adjusted health system quality based on workload beyond current resources, included the impact of public health efforts and notably considered how policy-makers could support such extraordinary efforts and how public solidarity could help or hinder efforts.

After two-days, ETC demonstrated a wide range of problems along with some successes. During the exercise, participants underscored that lessons learned from COVID-19 were not enough to respond to the unique challenges presented by a sustained mass casualty event. Despite this, key problems or challenges were predictable: the importance of clear leadership and the need for critical and human resources. Perhaps most importantly, the exercise surfaced several activities we could rapidly undertake now to be better prepared including considerations for repatriation and triage, patient registries, burn care, and blood supplies. These findings are explored in greater detail in Box 1.

**Box 1** Summary of selected ETC action areas to build resilience

**Table:** 

• Creation of a single repatriation hub: Given the need for coordinated repatriation and onward triage, Pearson International Airport’s shuttered Terminal 2, was proposed as a site for a single repatriation hub in Ontario • Artificial-Intelligence (AI)-enhanced triage: AI-enhanced triage has the potential to augment human assisted triage at every level of care (Biswas et al., 2023; Talley et al., 2024). Yet, more research and development is needed to better understand and evaluate its application to support more effective and efficient patient flows during a prolonged crisis. This aligns with NATO efforts exploring AI to provide optimized triage decision support along the patient evacuation pathway (Leone et al., 2024) • Pan-Canadian trauma registry: Establishing a trauma registry that links point of point of injury care to rehabilitation was emphasized as a way to bridge existing gaps between military and civilian data systems. The trauma registry will be necessary to conduct performance improvement and patient safety initiatives that minimize unnecessary strain on definitive care centres by quickly identifying changes in injury, characteristics, treatments and outcomes to allow for the development of clinical guidelines and new policies (Caminsky & Wong, 2023; Eastridge et al., 2009). By having a complete picture of care from point of injury to rehabilitation, this will allow the trauma system to function effectively and efficiently to minimize mortality, morbidity, and excessive demands upon the healthcare system • Strengthening burn care: ETC corroborated the demands a sustained mass casualty event would place on burn care across Canada (Gomez et al., 2025). An area already constrained by workforce and resource shortages, efforts are urgently needed to strengthen our burn care capacities, including building domestic manufacturing capacity for skin replacement for burn patients, given our current reliance on US production for cadaveric skin preparations (Gus et al., 2024; Kelly et al., 2021; Vrouwe et al., 2018) • Surge capacity for blood products: Canadian Blood Services is involved in planning for surge capability for blood products. However, supply chain issues may impact can ability to meet both the blood requirements of the military and the civil population if there was a supply chain shock. For example, Canada does not have any domestic producers of blood bags. Innovation is essential to ensure surge capacity for blood products is maintained and enhanced. For example, CAF is currently funding Canadian Blood Services to develop a dried plasma program for stockpiling of plasma that would be useful in battlefield conditions (Peng et al., 2024)	

The activities identified by participants differ from prior pandemic-focused preparedness tasks, which are often grounded in health-oriented solutions (e.g., such as public health and social measures or medical countermeasures) to provide offramps to time-limited crises. ETC highlighted that a conflict-driven mass casualty event demands that we adopt a different “whole of society” approach, one that engages the civil society in planning given ethical trade-offs inevitable in creating a staged approach with pre-identified triggers for activating wartime paradigm shifts in health services organization and delivery during an emergency of this nature. While the health sector cannot offer a way out of this type of crisis, we must prepare now to better absorb its impacts. Moreover, ETC’s recommendations are more specific and tactical than those in other preparedness guidance such as Health Canada’s pandemic influenza planning guidance for the health sector (Pan-Canadian Public Health Network, 2018). Part of this specificity may be due to the focused nature of the exercise. It looked at a specific source of health system strain (conventional warfare) within an all-hazards approach (captured with new challenges introduced at the end of each round), in one jurisdiction (Ontario). Part of the specificity of ETC’s recommendations may also result from the selection of participants and the setting. All participants were experts in their fields and with the opportunity to collaborate in real-time they were able to develop specific solutions. Moreover, with academic institutions as the hosts for ETC, it may have been easier for participants to propose, debate and refine solutions openly without the inevitable discussions about federal and provincial roles, transfer payments and other issues that can interfere with solution development. Finally, the wargame technique underlying the simulation may itself help produce more specific recommendations than the typical BOGGSATT (Bunch Of Guys and Gals Sitting Around the Table Talking) approach that defines a number of preparedness exercises.

Participants reflected that the exercise was an essential first step towards planning for a sustained mass-casualty situation and offered an opportunity to think broadly across the health sector; challenge silo-ed approaches when responding to a health crisis; and promote more integrated and coordinated preparedness planning. Pre- and post-exercise feedback surveys revealed marked positive changes in perception of preparedness for a sustained mass casualty event, notably in perception of our ability to coordinate between civilian and military health systems, as well as an increased understanding of the roles of organizations in responding to such a crisis. Perhaps most importantly, ETC created or strengthened the relationships and readiness to work together amongst those who would have to respond.

## Simulations

Tabletop exercises that simulate complex crises should be a routine part of building, assessing and enhancing our public health and health systems’ preparedness and resilience. These exercises complement ongoing public health risk prioritization and strategic foresight work to prepare for present and future threats to health and well-being across Canada. Tabletop exercises are an important way to build relationships amongst partners, encourage shared learning, and create space to identify innovative and contextualized solutions to respond to complex crises. These types of exercises centre the relationship-building that is essential to articulating the team-of-teams approach necessary to mount an effective response across public health and health systems. Importantly, they also create space for participants to do the necessary work of thinking the unthinkable to be better prepared. Thomas Schelling, the Nobel prize-winning godfather of game theory explained that “Games have one quality that separates them qualitatively from straightforward analysis and permits them to generate insights that could not be acquired through analysis, reflection, and discussion. That quality can be illustrated by the impossibility theorem: one thing a person cannot do, no matter how rigorous their analysis or heroic their imagination, is to draw up a list of the things that would never occur to them” (Schelling, 1986).

## Conclusions

Canada has overcome the challenges of previous global conflicts with determination and compassion, becoming a leader in health innovation for returning veterans. But old lessons from previous conflicts need to be relearned and updated for the new realities of twenty-first century state-on-state industrial warfare. Ad hoc reactions will not carry us through prolonged complex health crises. Successful navigation of such challenges is predicated on our national and collective capacity to prepare, respond and recover. Likewise, our responses will be smoother and more effective when they are practiced with warm hands on all side of the collaborations.

Our public health and health systems will continue to face crises and must withstand the broad challenges we face, whether external like the threat in ETC or internal like wildfires or floods that breach city limits. It is our collective responsibility, and an essential public health function, to be prepared for and meet these multidimensional threats on the horizon. We must be ready to protect and promote health and well-being, and care for the people who need it, when they need it, while minimizing social disruption. The challenges posed in ETC add a renewed moral duty to those productive citizens who volunteer to serve our country and risk their lives and health. Regular tabletop exercises like ETC should be a routine part of boosting our preparedness. Perhaps the best recommendation arising from ETC is the consistent request by already very busy participants to repeat ETC on an annual basis, create complementary exercises to test other areas of the system, and expand it to a pan-Canadian level. Successful collaboration between civilian and military stakeholders to create two mutually reinforcing layers of resilience is critical—our national security, stability, and prosperity may depend upon it.

## Data Availability

N/A.

## Code availability

N/A.
